# Co-infection with Dengue Virus and Pandemic (H1N1) 2009 Virus

**DOI:** 10.3201/eid1605.091920

**Published:** 2010-05

**Authors:** Eric Lopez Rodriguez, Kay M. Tomashek, Christopher J. Gregory, Jorge Munoz, Elizabeth Hunsperger, Olga D. Lorenzi, Jorge Gutierrez Irizarry, Carlos Garcia-Gubern

**Affiliations:** Hospital San Lucas/Ponce School of Medicine, Ponce, Puerto Rico, USA (E.L. Rodriguez, J.G. Irizarry, C. Garcia-Gubern); Centers for Disease Control and Prevention, San Juan, Puerto Rico, USA (K.M. Tomashek, C.J. Gregory, J. Munoz, E. Hunsperger, O.D. Lorenzi)

**Keywords:** Influenza, dengue, co-infection, viruses, pandemic (H1N1) 2009 virus, letter

**To the Editor**: Dengue is a mosquito-borne viral infection caused by 4 related dengue viruses. Each of these viruses is capable of causing classic dengue fever or dengue hemorrhagic fever (DHF), but may also cause nonspecific febrile illnesses. As a result, dengue is often difficult to diagnose clinically, especially because peak dengue season often coincides with that of other common febrile illnesses in tropical regions (*1*). Concurrent outbreaks of influenza and dengue have been reported (*2*,*3*); this circumstance often leads to delayed recognition of the presence of one or other disease in the community.  In April 2009, a new strain of influenza A virus known as pandemic (H1N1) 2009 virus was first detected in the United States (*4*). Pandemic (H1N1) 2009 infections were first detected in Puerto Rico in June 2009, and 59 deaths caused by the virus have been confirmed to date. This influenza outbreak coincided with the typical dengue season in Puerto Rico, which led to diagnostic difficulties; both infections disproportionately affected young persons, who often had similar, nonspecific symptoms.

We describe a case of laboratory-confirmed co-infection of dengue virus and pandemic (H1N1) 2009, and discuss the difficulties in distinguishing the 2 illnesses clinically. A 33-year-old woman (healthcare worker) in Ponce, Puerto Rico, sought treatment at an emergency department of a hospital in the southern part of the island with a 3-day history of febrile illness. Her symptoms began with throat irritation and earache; subsequently, cough, fever, and headache developed. She reported palpitations and generalized malaise but no other symptoms. The patient had no notable medical history and denied taking any medicines apart from over-the-counter antipyretics. She reported recent exposure to influenza at work and multiple recent mosquito bites. On physical examination, she had a temperature of 37°C, a heart rate of 91 bpm, and blood pressure of 125/82 mm Hg. A tourniquet test result was positive. Her pharynx was erythematous without exudate, and she had rhinorrhea. She had no lymphadenopathy, rash, petechiae, or purpura. Several small, red papules, which the patient described as recent mosquito bites, were on her legs. The remainder of her examination showed no unusual findings.

Laboratory studies showed a leukocyte count of 5,300 cells/mm^3^ with a normal differential count, hematocrit 35.2%, and thrombocyte count of 239,000 cells/dL. Results of a chest radiograph was unremarkable. A nasopharyngeal swab was positive for influenza A virus by rapid test. Nasopharyngeal and serum samples were sent to the Centers for Disease Control and Prevention (Dengue Branch) for influenza and dengue testing. The patient was diagnosed with suspected pandemic (H1N1) 2009 infection and prescribed oseltamivir for 5 days. She returned for a follow-up visit 12 days after the onset of symptoms. She reported having 2 more days of fever after her initial visit, but had no rash, petechiae, bleeding, or progression of respiratory symptoms. A second serum specimen was obtained during this visit.

The initial serum specimen was positive for dengue virus by serotype-specific, singleplex, real-time reverse transcription–PCR (*5*). Her nasopharyngeal specimen was positive by PCR for pandemic (H1N1) 2009 influenza. The second, convalescent-phase, serum specimen was negative for dengue immunoglobulin (Ig) M by IgM antibody–capture ELISA. The acute-phase and convalescent-phase samples were positive for IgG against dengue by ELISA (*6*), which indicated a secondary dengue infection. IgG titers exceeded the limits of the test for acute-phase and convalescent-phase samples, showing unusually elevated levels of IgG against dengue.

Distinguishing dengue and influenza by clinical features alone can be difficult. In an investigation of simultaneous dengue and influenza A outbreaks in Puerto Rico in 1977, similar percentages of persons with confirmed dengue and confirmed influenza had classic dengue symptoms (*2*). Hemorrhagic manifestations, like those typically seen in DHF, have been reported with influenza A in prior outbreaks (*7*,*8*) and with pandemic (H1N1) 2009 (Centers for Disease Control and Prevention, unpub. data). Previous influenza A outbreaks were initially believed to be outbreaks of DHF until careful laboratory investigation proved otherwise (*8*). Our patient did not have the typical signs and symptoms of dengue (rash, eye pain, thrombocytopenia, arthralgia, petechiae, or bleeding) that would differentiate her condition from that of patients with other febrile illnesses. She did have a positive tourniquet test result and fever, which have been advocated as screening criteria for dengue infection in children, at the time of initial examination (*9*). Data for the specificity and sensitivity of these criteria in adults are sparse, however, and some studies have shown a high incidence of positive tourniquet test results in patients with laboratory-confirmed influenza (*7*,*8*).

Our report demonstrates that co-infection with dengue virus and pandemic (H1N1) 2009 can occur. Previous studies also have shown cases of probable co-infection with seasonal influenza and dengue (*1*,*10*), including 1 fatal case (*1*). Because many dengue-endemic countries are experiencing pandemic (H1N1) 2009 outbreaks, providers should consider the possibility of viral co-infection, especially in severe cases, and should consider testing for both viruses.
